# Specific Genomic Fingerprints of Phosphate Solubilizing *Pseudomonas* Strains Generated by Box Elements

**DOI:** 10.1155/2014/496562

**Published:** 2014-12-15

**Authors:** Mohammad Bagher Javadi Nobandegani, Halimi Mohd Saud, Wong Mui Yun

**Affiliations:** Institute Tropical Agriculture, Universiti Putra Malaysia, 43400 Serdang, Selangor, Malaysia

## Abstract

Primers corresponding to conserved bacterial repetitive of BOX elements were used to show that BOX-DNA sequences are widely distributed in phosphate solubilizing *Pseudomonas* strains. Phosphate solubilizing *Pseudomonas* was isolated from oil palm fields (tropical soil) in Malaysia. BOX elements were used to generate genomic fingerprints of a variety of *Pseudomonas* isolates to identify strains that were not distinguishable by other classification methods. BOX-PCR, that derived genomic fingerprints, was generated from whole purified genomic DNA by liquid culture of phosphate solubilizing *Pseudomonas.* BOX-PCR generated the phosphate solubilizing *Pseudomonas* specific fingerprints to identify the relationship between these strains. This suggests that distribution of BOX elements' sequences in phosphate solubilizing *Pseudomonas* strains is the mirror image of their genomic structure. Therefore, this method appears to be a rapid, simple, and reproducible method to identify and classify phosphate solubilizing *Pseudomonas* strains and it may be useful tool for fast identification of potential biofertilizer strains.

## 1. Introduction

Biofertilizer industry is facing with the important challenge to identify potential strains of each species rapidly and precisely. In this regard, the* Pseudomonas* species have shown better performance comparing to others [1].* Pseudomonas* strains within the species cannot be reliably distinguished by their cellular metabolisms or other phenotypic characteristics [2–5]. Therefore, strains classification is mostly based on one or more host plants [6]. Based on the phosphate solubilizing ability, which is expressed in wide distribution in* Pseudomonas* species, this classification cannot be conclusive and is open to alternative interpretations [7–10]. Several attempts such as fatty acids profiling [11, 12], genomic and plasmid DNA analysis [10, 13–19], and protein analysis [5, 12, 20] have been used to classify strains and overcome this problem, even though these techniques are time-consuming, expensive, or sometimes sensitive to use in routine lab works. Thus, it could be useful to find the fast, rapid, and precise identification method to detect the most reliable and promising strains within the lot of strains which were detected as phosphate solubilizing* Pseudomonas* on the basis of genomic fingerprinting approach.

Families of repetitive DNA sequences which were dispersed throughout the whole genome of various bacterial species were studied recently [21, 22]. One hundred fifty-four base-pair sequences which were determined as BOX-element [23] repetitive DNA sequences have been studied in more detail. These repetitive DNA sequences play an important role with the potential to construct stem-loop structure in the organization of bacterial genome [21, 24, 25]. Bacterial genomic organization is thought to be shaped by selection; therefore, the distribution of BOX-elements' sequences can be indicative of the structure and evolution of the bacterial genome [21, 24, 25]. On the basis of this theory and the clonal nature and population dynamics of bacteria [9, 13, 18, 26–28] it can be theorized and assumed that each evolutionary line or strains have a unique distribution or arrangement of BOX repetitive sequences throughout the genomes and that enables us to generate specific genomic fingerprints of each isolate (strain).

In this paper, the ability of the PCR technique with the BOX-element corresponding primers to generate specific DNA fingerprints of phosphate solubilizing* Pseudomonas* species is demonstrated. Also, this technique can be a potential tool for identification of the phylogenic relationships between the best phosphates solubilizing* Pseudomonas* for biofertilizer industry application.

## 2. Materials and Methods

### 2.1. Bacterial Isolates

All phosphate solubilizing* Pseudomonas* isolates used in this study were isolated from the rhizosphere and nonrhizosphere of different locations (UPM-Semenyih-Dengkil oil palm fields) in Malaysia by using the modification method described by Nautiyal, 1999 [29]. The isolates have been systematically identified by 16S rRNA method as* Pseudomonas *sp. and are listed in Table 1. All isolates were stored at −80°C in glycerol stock and streaked on nutrients agar for further applications [30].

### 2.2. Bacterial DNA Preparation

Genomic DNA was extracted from isolated bacteria using a commercial kite (Qiagen Miniprep 27104 Matrix Technologies Cooperation, USA) according to the manufacturer's instructions. Bacteria cells were grown overnight at 28°C in LB broth with shaking. One milliliter of bacterial fresh culture was transferred to 1.5 mL microcentrifuge tube and centrifuged at 10,000 ×g for 4 minutes at 4°C. The supernatant was discarded and the bacterial pellet resuspended in 100 *μ*L 1x bactozym. Vortexing the mixture was resulted in a homogenous suspension, and then the mixture was incubated at 50°C for 30 minutes. Four hundred of DNAZOl solutions (TalronBiotech, USA) were added to the lysate bacterial suspension and then it was mixed for 30 seconds and then incubated at room temperature for 5 minutes. DNA was precipitated by adding 0.3 mL of 100% ethanol and mixed by inversion for 15 seconds and then stored at room temperature for 5 minutes. Then the samples were transferred into a column that was assembled in a clean collection tube provided by the company; the samples were centrifuged at 10,000 ×g for 1 minute. The column was washed with 750 *μ*L of washing buffer (provided by Qiagen kit) and centrifuged at 10,000 ×g two times for 1 minute each. Column was placed into the clean microcentrifuge tube and 50 *μ*L TE buffer was added directly onto column membrane and the mixture stood for 2 minutes. Again the tube was centrifuged at 10,000 ×g for 1 min to elute DNA. DNA was stored at −20°C.

### 2.3. PCR Amplification and Separation of DNA Bands

The primer sequences corresponding to BOX elements (41) (5′-CTACGGCAAGGCGACGCTGACG-3′) and 16S rDNA [31] (f5′-CCGAATTCGTCGACAACAGAGTTTGATCCTGGCTCAG-3′, and r5′-CCCGGATCCAAGCTTACGGCTACCTTGTTACGACTT-3′) were synthesized at BioSynTech Sdn Bhd (HICOM Glenmarie Industrial Park, 40150 Selangor DE, Malaysia). PCR condition for 16S rDNA that was used had been described by Weisburg et al., 1991 [31], by some modifications. PCR amplification for 16S rDNA was performed by thermal cycler with the following program: initial denaturation was at 95°C for 3 minutes, followed by 35 cycles consisting of denaturation at 94°C for 30 seconds, annealing at 60°C for 30 seconds, and elongation at 72°C for 2 minutes, and then the reaction was finished with an extension step at 72°C for 5 minutes. The BOX-PCR protocol and amplification were described by Versalovic et al., 1993 [22]. After the reactions, 8 to 10 *μ*L of the REP-PCR products was separated on 1% agarose gels, strained with ethidium bromide, and photographed by using gel-documentation system (Hoefer PS 500XT) [32].

### 2.4. Cluster Analysis (CA)

For cluster analysis (CA) of data, a matrix was used to generate a genetic distance. For that, Euclidean and Jaccard coefficient similarity matrix were used and then dendrogram of relationship was generated through unweighted pair-group method average (UPGMA) using the software package NTSYS-pc program [33].

## 3. Results

**Figure 1:**
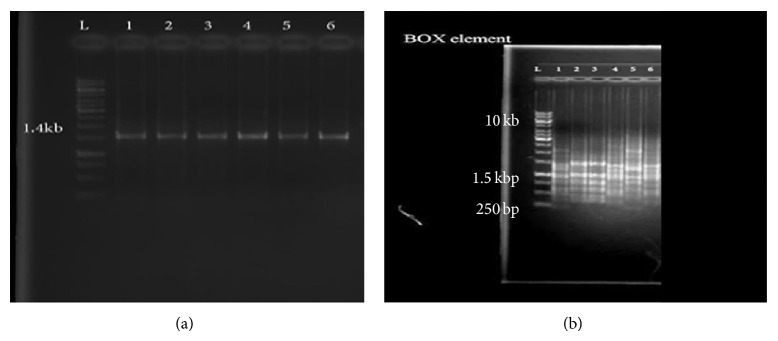
PCR products of 16S rDNA (a) and BOX element (b) (Line 1 = 18DNR, Line 2 = 5DNR, Line 3 = 41DNR, Line 4 = 22DNR, Line 5 = 31SR, and Line 6 = 8SR).

Two universal oligonucleotides were used to determine and identify the 16S rRNA gene for all isolates. The primers amplified the gene successfully from all phosphate solubilizing bacteria isolates. It was seen that there were not obvious variations in the size of rRNA gene products between the six isolated bacteria and the size of the 16S rRNA gene product of all isolated bacteria investigated in this study was approximately 1.4 Kb to the relative DNA size marker (Figure 1(a)).

Comparing the partial 16S rDNA sequence from the six bacterial isolates with sequences from the data base (NCBI) showed that they belong to the gamma subdivision of Proteobacteria phylum. 18DNR, 41DNR, 22DNR, 31SR, and 8SR bacterial isolates were classified in* Pseudomonas* genus as a* Pseudomonas *sp.; however 5DNR was identified as a* Pseudomonas fluorescens*. Sequences from these isolates were 98% or more similar to other 16S rDNA sequences from data base (Table 1).

**Figure 2:**
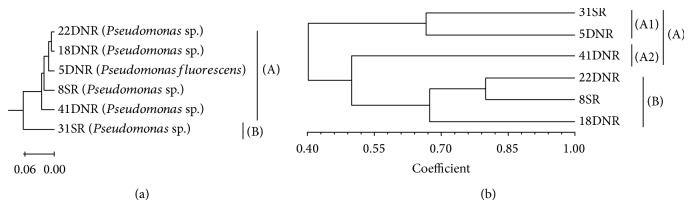
Dendrogram of phosphate solubilizing* Pseudomonas* bacteria based on the 16S rDNA (a) and BOX-PCR marker (b).

The phylogenetic analysis based on the partial 16S rDNA sequencing was able to discriminate the two main taxonomic lineages using DNA neighbor phylogenetic tree program (Figure 2(a)). Within the main lineage, the sequences obtained from the bacterial strains associated with 31SR were formed in the branch separated from the sequences of other bacteria that were isolated from soil. This feature was clear within the branch enclosing the sequences belonging to other isolates. There were six phylogeny branches that belonged to* Pseudomonas* strains and they showed more than 99.094% similarity with each other (Figure 2(a)).

Neighbor-joining analysis revealed the presence of two well resolved lineages according to 16S rDNA sequence analysis: designated clusters (A) and (B) (Figure 2(a)). Cluster (A) included 18DNR, 41DNR, 22DNR, 8SR, and 5DNR bacterial isolates. The similarity between them was more than 99.96%. Cluster (B) included the one species of* Pseudomonas *sp. (31SR). Gene sequences of 16S rDNA from phosphate solubilizing* Pseudomonas* bacteria were grouped within gamma proteobacteria.

### 3.1. BOX-PCR Analysis

PCR fingerprints with the BOX element primer (BOX-PCR) revealed species-specific band patterns for the various isolates of phosphate solubilizing* Pseudomonas*. DNA fingerprints obtained from BOX-PCR of extracted genomic DNA yielded comparable patterns. BOX-PCR as a precise molecular marker allowed better discrimination than 16S rDNA sequencing between strains within* Pseudomonas* species (isolates). BOX-PCR of these isolates revealed six different fingerprint profiles among six isolates of phosphate solubilizing bacteria isolated from oil palm soil (Figure 1(b)).

PCR with BOX-PCR primer and chromosomal DNA from the strains yielded multiple distinct DNA products of sizes ranging from approximately 300 to 5000 bp. The BOX-PCR patterns of* Pseudomonas* species designates were found to be highly related to one another (>40%). 18DNR and 22DNR and 8SR* Pseudomonas* bacterial isolates which were identified as a* Pseudomonas *sp.were found to be highly related to one another (>70%) but very distinct from 41DNR (*Pseudomonas *sp.), 5DNR (*Pseudomonas fluorescens*), and 31SR (*Pseudomonas *sp.) (Figure 2(b)).

The BOX-PCR marker similarities using Jaccard's coefficient were calculated using the data analysis. The matrix of coefficient was then used to cluster the similar accession based on BOX-PCR data and then to construct the dendrogram of relationship through the UPGMA. The dendrogram showing the genetic relationship of the isolates is presented in Figure 2(b).

The cluster analysis of* Pseudomonas* species based on the BOX-PCR identified two major groups ((A), (B)) at genetic distance = 0.60 (Figure 2). Cluster (A) contained three isolates, with the calculation of different locations (Semenyih, Dengkil): isolates 31SR, 5DNR, and 41DNR within this cluster. In cluster (A) there was two subclusters which included (A1) (31SR, 5DNR) and (A2) (41DNR). Cluster (B) was formed by three isolates. In this cluster isolates 22DNR, 8SR, and 18DNR were very similar to each other, based on the 16S rDNA sequencing; however, there was less than 70% similarity based on the BOX-PCR. It could reveal that they were different strains. Clusters (A) and (B) together formed a main cluster at genetic distance 0.4.

## 4. Discussion

In this study we have demonstrated that BOX elements as repetitive sequences were present in the genome of phosphate solubilizing* Pseudomonas* isolates, confirming and extending the conclusion of Versalovic et al., 1991 [34], and Akkermans et al., 1995 [35], and these sequences are virtually ubiquitous. We have also demonstrated that the BOX-PCR protocols were particularly suitable for the rapid molecular characterization of phosphate solubilizing* Pseudomonas* bacteria, especially at the strains level. The BOX-PCR protocol clearly had the potential to differentiate between isolates, including those that were not easily distinguished by other phenotypic and phylogenetic techniques such as 16S rDNA.

The data presented here suggested that BOX-PCR could be a useful tool for identification of phosphate solubilizing* Pseudomonas* purposes in industrial biofertilizers technology. Similar outcomes have been made about the utility of BOX-PCR in human pathology [22, 36, 37].

Several circumstances must be considered if BOX-PCR is to be useful for the proper identification of unknown phosphate solubilizing* Pseudomonas* isolates. Firstly, the characteristic of BOX-like sequences on gel and therefore the genomic fingerprint patterns generated by BOX-PCR must be established over time and distance. Comparison of the genomic fingerprint profiles of phosphate solubilizing* Pseudomonas* isolates within a tropical areas separated by time or distance supports the idea that the profiles remain stable. This similarity of fingerprint profiles of isolates by time has been noted by others too [37]. Secondly, the BOX-PCR technique must be able to distinguish among related bacterial strains with sufficient declaration and it, also, should be reproducible.

### 4.1. Supporting the Reproducibility of the BOX-PCR Protocol

Large numbers of phosphate solubilizing* Pseudomonas* isolates have determined that there was homogeneity of fingerprint profiles within each isolate. It was systematically shown that BOX in general could determine that the differences between phosphate solubilizing* Pseudomonas* isolates and substantial polymorphism were detected. Therefore, the distribution of BOX sequences was an accurate reflection of genomic structure.

Other PCR-based genomic fingerprinting techniques have been described and demonstrated to use for differentiating bacteria and diagnostic purposes [38–42]. There are some fundamental differences between BOX-PCR and RAPD-DNA analyses. The major difference lies in the length of the primers and the consequent PCR conditions. RAPD analysis relies on the use of primers with arbitrary sequences [43, 44], whereas BOX-PCR involves the use of primers of 22 bp with high homology to repetitive sequences [34]. The BOX primer permits the use of more inflexible PCR conditions, which in turn reduce experimental variation and PCR artefacts.

As noted by others, the BOX-PCR technique is very useful for bacterial strain identification; however, the utility of that for bacterial taxonomy may be limited to closely related strains [36, 45, 46]. Protein profile analysis or fatty acid profile analysis [12], serologic testing [47], and rRNA gene restriction patterns [10] support the distinctiveness of BOX-PCR analysis.

The BOX-PCR fingerprint profiles between the two isolates contain many bands of equal mobility and rely on the concept that selection for a specialized niche affects genome organization [25] and that corresponds to a unique distribution of repetitive sequences in the bacterial genome.

The BOX-PCR of phosphate solubilizing* Pseudomonas* isolates technique may be limited to phylogenetic analysis, and it effectively differentiates between two evolutionary lines classified within the same taxon.

## 5. Conclusion

In conclusion, we have found that BOX sequences were widespread in phosphate solubilizing* Pseudomonas* isolates and could be used to generate genomic fingerprints within* Pseudomonas* species. Unique fingerprint profiles generated by BOX-PCR could be exploited for identification purposes and for discerning evolutionary lines of phosphate solubilizing* Pseudomonas* in oil palm fields. Revealing the population diversity of phosphate solubilizing* Pseudomonas* isolates, in turn, had implications for the implementation of them for biofertilizers industry programs, disease management strategies, and ecological and epidemiological studies.

## Figures and Tables

**Table 1:** Phosphate solubilizing *Pseudomonas* isolates.

Number	Isolate	Name	Accession number (NCBI)	Phylum	Class	Order	Family	Genus
1	18DNR	*Pseudomonas* sp.	KJ748597	Proteobacteria	Gammaproteobacteria	Pseudomonadales	Pseudomonadaceae	*Pseudomonas *
2	5DNR	*Pseudomonas fluorescens *	KJ748598	Proteobacteria	Gammaproteobacteria	Pseudomonadales	Pseudomonadaceae	*Pseudomonas *
3	41DNR	*Pseudomonas* sp.	KJ783454	Proteobacteria	Gammaproteobacteria	Pseudomonadales	Pseudomonadaceae	*Pseudomonas *
4	22DNR	*Pseudomonas* sp.	KJ729599	Proteobacteria	Gammaproteobacteria	Pseudomonadales	Pseudomonadaceae	*Pseudomonas *
5	31SR	*Pseudomonas* sp.	KJ748596	Proteobacteria	Gammaproteobacteria	Pseudomonadales	Pseudomonadaceae	*Pseudomonas *
6	8SR	*Pseudomonas* sp.	KJ783451	Proteobacteria	Gammaproteobacteria	Pseudomonadales	Pseudomonadaceae	*Pseudomonas *
